# Alterations of Growth and Focal Adhesion Molecules in Human Breast Cancer Cells Exposed to the Random Positioning Machine

**DOI:** 10.3389/fcell.2021.672098

**Published:** 2021-06-30

**Authors:** Jayashree Sahana, Thomas J. Corydon, Markus Wehland, Marcus Krüger, Sascha Kopp, Daniela Melnik, Stefan Kahlert, Borna Relja, Manfred Infanger, Daniela Grimm

**Affiliations:** ^1^Department of Biomedicine, Aarhus University, Aarhus, Denmark; ^2^Department of Ophthalmology, Aarhus University Hospital, Aarhus, Denmark; ^3^Department of Microgravity and Translational Regenerative Medicine, Clinic for Plastic, Aesthetic and Hand Surgery, Otto von Guericke University, Magdeburg, Germany; ^4^Research Group “Magdeburger Arbeitsgemeinschaft für Forschung unter Raumfahrt- und Schwerelosigkeitsbedingungen” (MARS), Otto von Guericke University, Magdeburg, Germany; ^5^Institute of Anatomy, Otto von Guericke University, Magdeburg, Germany; ^6^Department of Radiology and Nuclear Medicine, Experimental Radiology, Otto von Guericke University, Magdeburg, Germany

**Keywords:** microgravity, breast cancer, multicellular spheroids, focal adhesions, cytoskeleton, extracellular matrix

## Abstract

In this study, we evaluated changes in focal adhesions (FAs) in two types of breast cancer cell (BCC) lines (differentiated MCF-7 and the triple-negative MDA-MB-231 cell line) exposed to simulated microgravity (s-μ*g*) created by a random positioning machine (RPM) for 24 h. After exposure, the BCC changed their growth behavior and exhibited two phenotypes in RPM samples: one portion of the cells grew as a normal two-dimensional monolayer [adherent (AD) BCC], while the other portion formed three-dimensional (3D) multicellular spheroids (MCS). After 1 h and 30 min (MDA-MB-231) and 1 h 40 min (MCF-7), the MCS adhered completely to the slide flask bottom. After 2 h, MDA-MB-231 MCS cells started to migrate, and after 6 h, a large number of the cells had left the MCS and continued to grow in a scattered pattern, whereas MCF-7 cells were growing as a confluent monolayer after 6 h and 24 h. We investigated the genes associated with the cytoskeleton, the extracellular matrix and FAs. *ACTB, TUBB, FN1, FAK1*, and *PXN* gene expression patterns were not significantly changed in MDA-MB-231 cells, but we observed a down-regulation of *LAMA3, ITGB1* mRNAs in AD cells and of *ITGB1, TLN1* and *VCL* mRNAs in MDA-MB-231 MCS. RPM-exposed MCF-7 cells revealed a down-regulation in the gene expression of *FAK1, PXN, TLN1, VCL* and *CDH1* in AD cells and *PXN, TLN* and *CDH1* in MCS. An interaction analysis of the examined genes involved in 3D growth and adhesion indicated a central role of fibronectin, vinculin, and E-cadherin. Live cell imaging of eGFP-vinculin in MCF-7 cells confirmed these findings. β-catenin-transfected MCF-7 cells revealed a nuclear expression in 1*g* and RPM-AD cells. The target genes *BCL9, MYC* and *JUN* of the Wnt/β-catenin signaling pathway were differentially expressed in RPM-exposed MCF-7 cells. These findings suggest that vinculin and β-catenin are key mediators of BCC to form MCS during 24 h of RPM-exposure.

## Introduction

Tumor diseases are a heavy burden for people around the world with high morbidity and mortality. The second leading cause of death worldwide is cancer (Sung et al., 2021). According to data published by the WHO Global Cancer Observatory (GLOBOCAN) in 2020, tumor diseases were responsible for an estimated 10 million deaths in 2020 (Sung et al., 2021).

In this study, we focused on breast cancer cells. Breast cancer (BC) is the most commonly diagnosed malignant tumor with an estimated 2.3 million new cases (11.7%) (Sung et al., 2021). BC is a very heterogeneous disease, as tumor cells can show different gene expression patterns (van’t Veer et al., 2002). An individualized therapy for patients is therefore necessary. BC shows seven molecular subtypes with typical histology, treatment options, and outcomes (van’t Veer et al., 2002; Nassef et al., 2019a). Patient survival rates are associated with the size of the tumor, the hormone receptor profile, and metastases at the time of diagnosis. Standard therapy is the surgical resection of the cancer. Depending on tumor type, size, staging, and metastasis to sentinel lymph nodes, strategies like chemotherapy, radiation and anti-hormone therapy, targeted treatment against HER2, and anti-angiogenic therapy are often applied after surgery or in cases of advanced disease stage (Kristensen et al., 2014). Despite advanced therapy, it is estimated that about 684,996 deaths worldwide will occur from BC per year (Sung et al., 2021). Therefore, novel therapeutic strategies with the help of new technologies are required.

Microgravity (μ*g*)-based cancer research currently generates major interest (Becker and Souza, 2013; Krüger et al., 2019). Gravitational biology and space medicine are growing areas of research worldwide. It is known today that cells cultivated in real and simulated μ*g* show changes in proliferation, transcription factors, gene expression patterns, and in the content of various proteins (Lewis et al., 1998; Boonyaratanakornkit et al., 2005; Chang and Hughes-Fulford, 2009; Pietsch et al., 2010; Chang et al., 2012; Svejgaard et al., 2015; Kopp et al., 2016, 2018; Buken et al., 2019; Grimm et al., 2020). Exposure of cells to real μ*g* and the random positioning machine (RPM) leads to changes in the cytoskeleton, focal adhesion components, extracellular matrix proteins, and differentiation, as well as to an increase in apoptosis and changes in growth behavior (Lewis et al., 1998; Svejgaard et al., 2015; Kopp et al., 2018; Buken et al., 2019; Nassef et al., 2019a,b).

In earlier investigations, MCF-7 cells (Soule et al., 1973) showed variable growth behavior when exposed to the RPM for one and 5 days: one portion of the cells remained adherent, while the second portion grew in the form of spheroids with and without gland-like structures (Kopp et al., 2016). In contrast, MCF-7 cells only formed compact spheroids when cultured for 24 h under conditions of simulated μ*g* created by an RPM (Kopp et al., 2016).

Our second cell line of interest is the well-described basal-like triple-negative MDA-MB-231 cell line, which has also been investigated in real μ*g* and on an RPM (Masiello et al., 2014; Nassef et al., 2019a). Exposure to simulated (s-) μ*g* resulted in three-dimensional (3D) growth and demonstrated a fundamental role in shaping form and function in human BCC (Masiello et al., 2014). These cell cultures in μ*g* can be performed scaffold-free to engineer 3D aggregates, including multicellular spheroids (MCS), tissues, and tubular structures (Grimm et al., 2020). The organoids can be used for research in space medicine and for testing the delivery and efficacy of drugs (Grimm et al., 2020). Cancer cells are sensitive to mechanical stress. The regulation of focal adhesions (FAs) and cytoskeletal dynamics are important for cell maintenance, cell adherence, cell movement, and migration (Hughes-Fulford, 2003; Li et al., 2009). It has been demonstrated that s-μ*g* reduces FAs and alters the cytoskeleton and nuclear positioning, leading to enhanced apoptosis in B16 melanoma cells (Zhao et al., 2018). Another study showed that the inhibition of FAs leads to reduced melanoma cell proliferation and metastasis (Tan et al., 2018).

Therefore, the objective of this study was to (i) investigate the changes and growth of BC cells exposed to s-μ*g* created by an RPM, (ii) and to test, based on previous findings, the hypothesis that 24 h of exposure to s-μg cells induces the formation of MCS and alters the expression of genes related to the cytoskeleton, cell adhesion, and focal adhesions (FAs) in BC. Finally, we focused on the migration behavior and invasion potential of the MCS of both BC cell types.

## Materials and Methods

### Cell Culturing and Microgravity Simulation on the RPM

MDA-MB-231 human breast adenocarcinoma cells (MDA-MB-231; ATCC^®^ HTB-26^TM^) and the MCF-7 human breast adenocarcinoma cell line MCF7 (ATCC^®^ HTB-22TM) were purchased from the American Type Culture Collection (Manassas, Virginia, United States). The cells were cultured in RPMI 1640 (Life Technologies, Naerum, Denmark) in complete medium supplemented with 10% fetal calf serum (Sigma-Aldrich St. Louis, MO, United States) and 1% penicillin/streptomycin (Life Technologies, Carlsbad, CA, United States) under standard cell culture conditions (37°C and 5% CO_2_). Three days prior to the experiment, 2 × 10^6^ cells were counted and seeded into T75 cm^2^ vented cap cell culture flasks (Sarstedt, Nümbrecht, Germany). A total number of 24 T25 cm^2^ cell culture flasks was prepared for the RPM experiments. Twelve flasks were kept under normal gravity conditions in the same incubator where the RPM was situated and used as ground controls (1*g*), and 12 flasks were mounted on the RPM. In addition, 2 × 24 slide flasks (NuncTM, ThermoFisher Scientific, Denmark) were prepared for each condition, and 1 × 10^5^ cells were seeded in each 9 cm^2^ slide flask for immunofluorescence staining. Each flask was completely filled with medium, in a complete air bubble-free condition. The flasks were installed on the center frame of the RPM and run for 24 h. The 1*g* controls were stored inside the device in the same incubator at 37°C and with 5% CO_2_.

After 24 h, the RPM was stopped, and the BC cells were examined and photographed by phase contrast microscopy. For qPCR investigations, the medium from RPM flasks was transferred into 50 mL tubes and centrifuged at 4°C to collect the MCS. The MCS were washed in phosphate buffered saline (PBS) (Life Technologies), centrifuged again and after removal of the PBS stored in liquid nitrogen. For harvesting the adherent cells from 1*g* control and RPM cultures from the cell culture flask bottom, 10 mL ice-cold PBS was added to each T75 cm^2^ flask, and the cells were scraped off with a scraper. The cell suspension was collected and centrifuged at 4°C. The PBS was discarded, and the dry pellet was washed twice with PBS and afterward stored in liquid nitrogen for protein extraction and RNA isolation. For immunofluorescence staining, the medium was discarded and the cells were washed twice with PBS. Afterward, 2 mL 4% PFA (paraformaldehyde solution) was added to fix the cells for 45 min at room temperature. Then, the PFA was discarded, and 2 mL of PBS was added to the slide flasks, which were then stored in a refrigerator for future staining.

### Simulated Microgravity Conditions Created by a Random Positioning Machine

The RPM was purchased from Airbus, Defense, and Space (former Dutch Space, Leiden, the Netherlands) and used to simulate microgravity. The method was published earlier in detail (Borst and van Loon, 2008; Wuest et al., 2015). In short, the RPM attempts to nullify the gravity vector by continuously rotating the central frame (where samples are mounted) around two perpendicular axes. The degree of rotation was in between 60°/s and 75°/s and randomized around both axes by continuously changing the direction; thus, the net magnitude of gravity vector toward the sample approaches zero, resulting in simulated microgravity. Twelve T25 cm^2^ flasks (12 each run) were fixed on the central frame of the RPM. After 24 h, the device was stopped, and the samples were collected and investigated by phase contrast microscopy. Three different groups were collected from the RPM experiments: 1*g*, AD, and MCS.

### Phase Contrast Imaging

The morphology of the 1*g* control and RPM BC cells before, during, and after the experiment were photographed using phase contrast microscopy. The images were taken with a Canon EOS550D camera (Canon GmbH, Krefeld, Germany) through a Leica DM IL LED inverted microscope (Leica Microsystems, Brønshøj, Denmark).

### Proliferation, Migration, and Invasion

Ki-67 immunofluorescence staining was performed to visualize the proliferating cells in MCS after 24 h. The immunofluorescence method is described in Section “Immunofluorescence Staining and Confocal Laser Scanning Microscopy.” To demonstrate the migration behavior of the MCS, the spheroids were collected after 24 h, pipetted in slide flasks and studied for adherence and migration by phase contrast microscopy (Leica Microsystems).

To test the invasion potential of the MCS of both cell lines, we cultured a confluent monolayer of human endothelial cells [EA.hy926 cell line (ATCC CRL-2922)] in slide flasks [detailed cell culture method published in reference (Dittrich et al., 2018)], pipetted MCF-7 or MDA-MB-231 BC MCS cells on the endothelial cells, and studied the invasion potential of the MCS cells. The examination was performed by phase contrast microscopy.

### Immunofluorescence Staining and Confocal Laser Scanning Microscopy

The BC cells investigated in slide flasks were fixed with 4% PFA solution for 30 min. Then, they were subjected to membrane permeabilization with 0.1% Triton X for 10 min and blocking with 1% [weight (w)/volume (v)] bovine serum albumin (BSA, Sigma-Aldrich, Steinheim, Germany) in PBS for 30 min. The slides were then released from the flasks, and the cells were incubated with primary antibodies (listed in Table 1) in a PBS solution with 1% (w/v) BSA overnight at room temperature. The next day, the cells were washed three times with PBS before incubation with the secondary Alexa Fluor 488 (AF488)-conjugated anti-rabbit (Cell Signaling Technology, Danvers, MA, United States) or anti-mouse antibody (Invitrogen) at a dilution of 1:1,000 for 2 h at ambient temperature. Afterward, the slides were washed with PBS three times and prepared and covered for microscopy using Fluoroshield^TM^ mounting media with DAPI (4′,6-diamidino-2-phenylindole; Sigma-Aldrich). The covered slides were kept at 4°C in a dark box until confocal microscopy was performed (Buken et al., 2019).

**TABLE 1:** List of antibodies used for immunofluorescence.

**Antibody**	**Immunofluorescence**	
E-cadherin	CST#2500S	Rb
Fibronectin	Millipore Sigma/F3648	Rb
Ki 67	Abcam# ab15580	Rb
Laminin	Sigma/L9393	Rb
Talin	Millipore Sigma/T3287	Ms
Vinculin	Millipore Sigma/V9131	Ms

*Ms, mouse; Rb, rabbit; Sigma (now by MERCK, Darmstadt, Germany); ABCAM (Cambridge, United Kingdom); Cell Signaling Technology (CST, BioNordika Denmark A/S, Herlev, Denmark**);** and MERCK Millipore (Burlington, Massachusetts, United States).*

Fluorescence staining was analyzed using a ZEISS LSM 710 CLSM (ZEISS, Jena, Germany) and a 40 × oil-immersion objective with a NA of 1.3 (Nyegaard et al., 2015; Corydon et al., 2016b). The quantification of the fluorescence intensity was performed with the FIJI image processing software (available from^1^). Briefly, data in.czi-format was imported using the Bio-Formats plugin and all color channels were split into separate grayscale images. In the green channel, individual cells were outlined with the freehand ROI tool and both area and integrated density was measured. Subsequently, background and secondary antibody control intensities were subtracted to yield the corrected total cell fluorescence. For each experimental condition, at least five different fields of view with a mean number of five cells were analyzed.

### RNA Isolation and Quantitative Real-Time Polymerase Chain Reaction

The method was published earlier in detail (Ma et al., 2013; Kopp et al., 2015). RNA isolation was performed using a RNeasy Mini Kit (Qiagen, Hilden, Germany) with an additional DNase digestion step (Qiagen) in order to eliminate residual DNA contamination. Afterward, the amount of RNA was quantified using a Photometer Ultrospec2010 (Amersham Biosciences, Freiburg, Germany). A first strand cDNA synthesis kit (Thermo Fisher Scientific, Waltham, MA, United States) was applied for reverse transcription. qPCR was performed on a 7,500 Fast Real-Time PCR System using the FAST SYBR Green Master Mix (both Applied Biosystems, Darmstadt, Germany) according to standard protocols (Kopp et al., 2016, 2018; Buken et al., 2019; Nassef et al., 2019a).

All samples were measured in triplicate. For normalization, 18S rRNA was used as a housekeeping gene. The comparative Ct (ΔΔCt) method was used for relative quantification of transcription levels, and the 1*g* control group was defined as 100% for reference. Before performing qPCR, primers were designed using NCBI Primer Blast, and they were selective for cDNA by spanning exon–exon junctions and had a melting temperature of around 60°C. The primers were synthesized by TIB Molbiol (Berlin, Germany) and are listed in Table 2:

**TABLE 2:** List of all the primers’ sequences used in the quantitative PCR. All the sequences are listed in the 5′-3′-direction.

**Factor**	**Primer Name**	**Sequence 5′–3′**
*18S*	18S-F	GGAGCCTGCGGCTTAATTT
	18S-R	CAACTAAGAACGGCCATGCA
*ACTB*	ACTB-F	TGCCGACAGGATGCAGAAG
	ACTB-R	GCCGATCCACACGGAGTACT
*BCL9*	BCL9-F	CAGAGCAGACAATAGGCCCC
	BCL9-R	AGACCCTTTTCCCGCAATCC
*CCND1*	CCND1-F	CCCTGACGGCCGAGAAG
	CCND1-R	AGGTTCCACTTGAGCTTGTTCAC
*CDH1*	CDH1-F	GCTGGACCGAGAGAGTTTCC
	CDH1-R	CAGCTGTTGCTGTTGTGCTT
*CTNNA1*	CTNNA1-F	AATTTAGCGCTCGCCCAG
	CTNNA1-R	ACAAGGGTTGTAACCTGTGTAA
*CTNNB1*	CTNNB1-F	GAAACAGCTCGTTGTACCGC
	CTNNB1-R	ATCCACTGGTGAACCAAGCA
*FAK1/PTK2*	FAK1-F	TGTGGGTAAACCAGATCCTGC
	FAK1-R	CTGAAGCTTGACACCCTCGT
*FN1*	FN1-F	AGATCTACCTGTACACCTTGAATGACA
	FN1-R	CATGATACCAGCAAGGAATTGG
*ITGB1*	ITGB1-F	GAAAACAGCGCATATCTGGAAATT
	ITGB1-R	CAGCCAATCAGTGATCCACAA
*JUN*	JUN-F	GAGCTGGAGCGCCTGATAAT
	JUN-R	CCCTCCTGCTCATCTGTCAC
*LAMA3*	LAMA3-F	AAAGCAAGAAGTCAGTCCAGC
	LAMA3-R	TCCCATGAAGACCATCTCGG
*MYC*	MYC-F	GGATTCTCTGCTCTCCTCGAC
	MYC-R	CTTCTTGTTCCTCCTCAGAGTC
*NFATC2*	NFATC2-F	CAAGACGAGCTTGACTTCTCCA
	NFATC2-R	GCATTCGGCTCTTCTTCGTTC
*PXN*	PXN-F	CATGGACGACCTCGACGC
	PXN-R	CAAGAACACAGGCCGTTTGG
*TBP*	TBP-F	GTGACCCAGCATCACTGTTTC
	TBP-R	GCAAACCAGAAACCCTTGCG
*TLN1*	TLN1-F	GATGGCTATTACTCAGTACAGACAACTGA
	TLN1-R	CATAGTAGACTCCTCATCTCCTTCCA
*TUBB*	TUBB-F	CTGGACCGCATCTCTGTGTACTAC
	TUBB-R	GACCTGAGCGAACAGAGTCCAT
*VCL*	VCL-F	GTCTCGGCTGCTCGTATCTT
	VCL-R	GTCCACCAGCCCTGTCATTT

### STRING Analysis

The interactions between proteins were determined using the STRING v10 platform (Snel et al., 2000). For each protein, the UniProtKB entry number was inserted in the input form “multiple proteins,” and “Homo sapiens” was selected as the organism. The resulting network view was downloaded in the molecular action view showing lines between interacting proteins and genes (Nassef et al., 2019a).

### Transfection of the MCF-7 Cell Line to Visualize Vinculin and β-Catenin

The MCF-7 BCC cells were stably transfected using a Sleeping Beauty (SB) transposon-based vector containing the eGFP-hVCL1 cassette for the visualization of vinculin as described in Nassef et al. (2019b). In brief, an eGFP-hVCL fragment containing NotI and XbaI restriction sites in the 5′ and 3′ ends, respectively, was sub-cloned into a Sleeping Beauty (SB) transposon-based vector pT2/CMV-linker-SV40-Neo (Staunstrup et al., 2011; Pihlmann et al., 2012) containing a linker, enabling insertion of the *Not*I-eGFP-hVCL1-*Xba*1 fragment. The resulting plasmid, pT2/CMV-eGFP-hVCL1-SV40-Neo, was entitled pSB-eGFP-vinculin. In order to provide stable expression of the eGFP-vinculin expression cassette, MCF-7 cells were co-transfected with pSB-eGFP-vinculin and pCMV−SB100X (Pihlmann et al., 2012) using X-tremeGENE 9 transfection reagent (Roche, Mannheim, Germany) according to the manufacturer’s protocol (Askou et al., 2015). An inactive SB transposase (mSB) was included as negative control. Transfected cells were cultured in medium containing G418 (geneticin) to allow for growth of stably transfected cells only. A fluorescence microscope was used to validate the efficiency of the transfection.

To visualize catenin in MCF-7 BCC cells, a cDNA fragment encoding human β-catenin N-terminally tagged with the mCherry marker protein was cloned into the piggyBac plasmid pPBT-CMV-MCS-IRES-Puro (Sharma et al., 2012). The resulting plasmid, entitled pPBT-mCherry-catenin, was transiently transfected into MCF-7 cells using X-tremeGENE 9 transfection reagent (Roche) according to manufacturer’s protocol (Askou et al., 2015). Following transfection, the cells were maintained for 24 h at 1*g* conditions and then transferred to either 1*g* or to the RPM for additional 24 h.

Images of MCF-7 BCC cells expressing eGFP-vinculin were captured by a confocal scanning microscope (CLSM) 710 (Zeiss, Jena, Germany). Images of MCF-7 BCC cells expressing mCherry-catenin were captured 48 h post-transfection by a CLSM 800 (Zeiss).

### Statistical Analysis

All statistical evaluations were performed using IBM SPSS Statistics 23 (IBM Deutschland GmbH, Ehningen, Germany). The Mann–Whitney U-Test was utilized to evaluate the statistical significance in the changes in expression levels following RPM exposure, thus comparing the 1g control to AD and MCS. A significance level of 0.05 was used. The standard deviation was calculated and presented together with the mean values as percentages in bar plots.

## Results

### Morphology, Cell Growth, Migration and Invasion Behavior of the BCC

**FIGURE 1:**
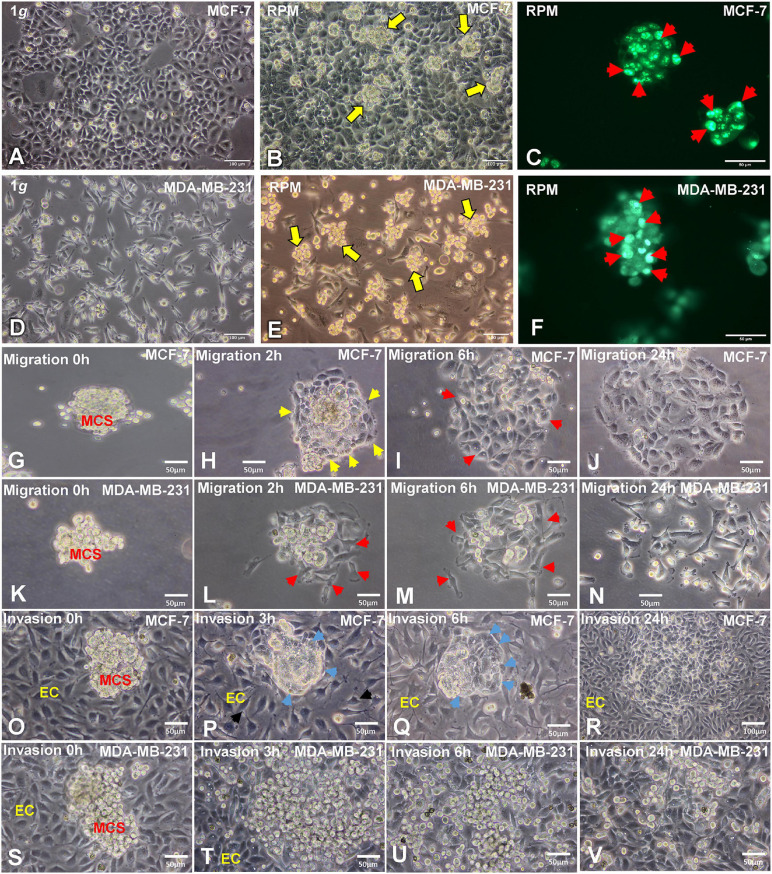
Phase contrast microscopy of MCF-7 and MDA-MB-231 breast cancer cells (BCC). **(A)** Characteristic epithelial morphology of the MCF-7 cell line cultured at 1*g*. **(B)** Adherent MCF-7 cells and multiple compact multicellular spheroids (MCS, yellow arrows) were visible when the MCF-7 cells were exposed to the random positioning machine (RPM) for 24 h. **(C)** Ki-67 immunofluorescence staining (IFS) of MCF-7 MCS. The red arrows show Ki-67 positive nuclei. **(D)** MDA-MB-231 cell line cultured under static 1*g* control conditions at 24 h. **(E)** MDA-MB-231 cells exposed to the RPM for 24 h. Multiple multicellular spheroids (yellow arrows) are visible as well as adherent cells. Magnification × 100. The yellow arrows indicate MCS floating in the supernatant of the cell cultures. **(F)** Ki-67 IFS of MDA-MB-231 MCS. The red arrows show Ki-67 positive nuclei. **(G)** Migration behavior of the MCF-7 cells in spheroids at 0 h, **(H)** 2h, the yellow arrows indicate the attachment of the MCS on the slide flask bottom and the cell membrane. **(I)** 6 h, the red arrows show areas of migration and **(J)** 24 h. **(K)** Migration behavior of MDA-MB-231 cells in spheroids at 0 h, **(L)** 2 h, the red arrows show the attached MCS and migration **(M)** 6 h, red arrows indicate migrated cells and after **(N)** 24 h. No MCS were visible at this time point. Invasion test: co-cultures of EA.hy926 endothelial cells and MCF-7 MCS. **(O)** 0 h, **(P,Q)** 3 h and 6h, the blue arrows show the formation of a cellular membrane. **(R)** After 24 h the MCF-7 BCC had invaded the endothelial cells. Invasion test: co-cultures of EA.hy926 endothelial cells and MDA-MB-231 MCS. **(S)** 0 h, **(T)** 3 h, at this time point the MDA-MB-231 cells had already migrated out of the MCS and showed an invasive growth. **(U)** 6 h and **(V)** 24 h.

MCF-7 and MDA-MB-231 BCC cultured under normal standard cell culture conditions at 1*g* grew as a two-dimensional (2D) monolayer (Figures 1A,D). MCF-7 revealed their characteristic epithelial growth behavior at 1*g* (Figure 1A). MDA-MB-231 cells exhibited an epithelial-like morphology and appeared phenotypically as spindle-shaped cells under normal 1*g* static conditions (Figure 1D). In contrast, both BCC types, the MCF-7 and MDA-MB-231 cells exposed to an RPM for 24 h showed two different phenotypes: adherent cells and multiple multicellular spheroids floating in the medium (Figures 1B,E).

The proliferation marker Ki-67 was determined to indicate the active phases of the cell cycle (G1, S, G2, and mitosis), because the Ki-67 protein is absent in resting G0 cells. The Ki-67 immunofluorescence showed various proliferating cells on the surface of the MCS of both cell types (Figures 1C,F).

In addition, we tested the migration behavior of the MCS. After 20 min 50% of the MCF-7 spheroids and 30% of the MDA-MB-231 spheroids were adherent to the slide flask bottom. All MDA-MB-231 MCS had attached after 1.5 h, and all MCF-7 MCS stayed adherent on the slide flask bottom after 1 h 40 min. Figures 1H,L show the MCS of both cell types at 2 h. The MDA-MB-231 cells started to migrate (Figure 1L), whereas the MCF-7 MCS exhibited an outer membrane (yellow arrows, Figure 1H). After 6 h, a large number of MDA-MB-231 BCC had migrated out of the MCS (Figure 1M) and only some remaining 3D aggregates are visible. In contrast, the MCF-7 MCS were still compact and only a few cells started to leave the MCS (red arrows, Figure 1I). In these areas, the membrane around the cells growing three-dimensionally was destroyed. After 24 h, MDA-MB-231 cells grew in form of a monolayer and the cells had lost their intercellular contacts (Figure 1N), whereas MCF-7 cells still grew as a more compact cell monolayer, but with a broader interstitial space (Figure 1J).

To evaluate the invasion behavior of both BC types, spheroids were seeded out on a confluent monolayer of EA.hy926 endothelial cells (ECs) (Figures 1O–V). After 3 h, MCF-7 spheroids exhibited an outer membrane (yellow arrows) at the contact area to the ECs. The ECs changed their orientation and provided more space for the tumor cells, which started their invasion (Figure 1P). The blue arrow shows the invasive MCF-7 cells, and the black arrow indicates the looser EC layer (Figure 1P). Three hours later, the MCF-7 MCS were invasive growing in between the ECs, providing space to the cancer cells (Figure 1Q). The overview given in Figure 1R shows the invasiveness of the MCF-7 tumor cells after a 24 h co-culture and their growth between the EC monolayer.

MDA-MB-231 spheroids seeded on ECs very rapidly attached and spread out onto the EC monolayer (Figure 1T). After a 6-h co-culture, all cells of the MCS had migrated out of the spheroid and progressed growing between the EC monolayer (Figure 1U). After 24 h, mostly single tumor cells were detectable between the ECs (Figure 1V).

### Changes in Cytoskeletal Factors in MDA-MB-231 Cells Exposed to the RPM

**FIGURE 2:**
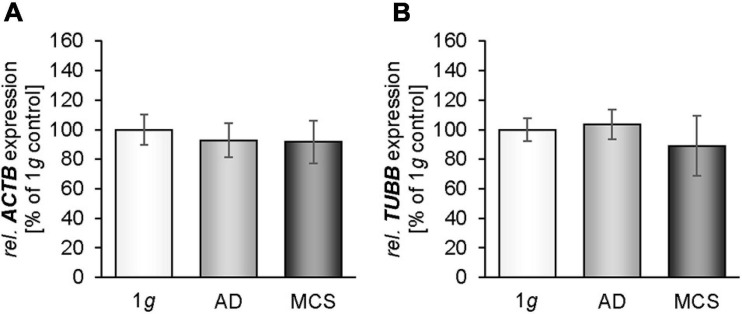
Gene expression [*ACTB* **(A)**, *TUBB* **(B)**] of MDA-MB-231 cells exposed to the RPM. * *p* < 0.05 1*g* vs. adherent (AD) and/or MCS and #*p* < 0.05 AD vs. MCS.

We measured the gene expression of β-actin in MDA-MB-231 cells exposed to the RPM. The *ACTB* mRNA expression was not altered in 1*g*, adherent (AD) and MCS samples (Figure 2A). In parallel, the *TUBB* mRNA expression was not changed in all groups (Figure 2B). The cytoskeletal components of MCF-7 cells were examined in an earlier study (Kopp et al., 2016).

### Changes in Cell Adhesion Molecules of MDA-MB-231 Cells Exposed to the RPM

**FIGURE 3:**
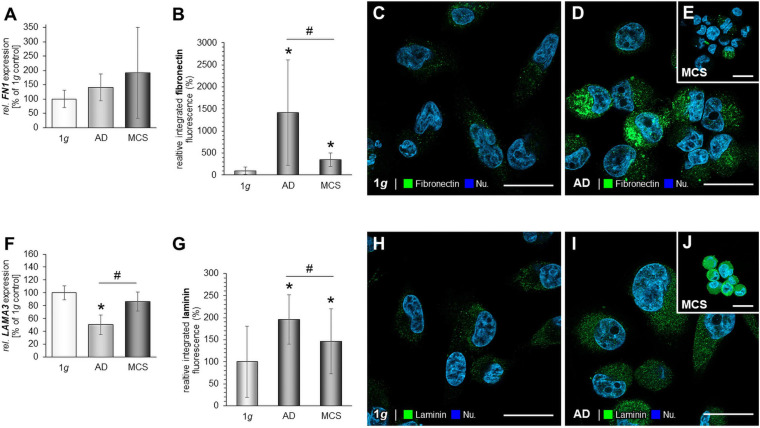
Investigation of fibronectin **(A–E)** and laminin **(F–J)**: The gene expression of *FN1* **(A)**, analysis of the fluorescence intensity of fibronectin **(B)** and immunofluorescence staining (IFS) of fibronectin in 1*g* samples of MDA-MB-231 cells: **(C)**, RPM-AD samples **(D)** and MCS **(E)**; the *LAMA3* mRNA expression **(F)** as well as analysis of the fluorescence intensity of fibronectin **(G)**, the IFS of laminin in 1*g* samples **(H)**, RPM-AD samples **(I)** and MCS **(J)** of MDA-MB-231 cells exposed to the RPM. *p < 0.05 1*g* vs. AD and/or MCS and #*p* < 0.05 AD vs. MCS.

We investigated the cell adhesion molecules fibronectin and laminin. There was no significant change in the gene expression of *FN1* in MCS after 24 h (Figure 3A). The fibronectin immunofluorescence was significantly elevated in RPM-AD cells and MCS compared with 1*g* cells (Figures 3B–E). In addition, the mRNA expression of *LAMA3* was not changed in MCS vs. 1*g*, but significantly down-regulated in AD cells in comparison to 1*g* static control cells (Figure 3F). The laminin immunofluorescence was increased in RPM-AD cells and MCS (Figures 3G–J). Both cell adhesion molecules and extracellular matrix proteins were investigated earlier in MCF-7 cells (Kopp et al., 2016).

### The Impact of RPM-Exposure on Focal Adhesions and Interacting Factors in MDA-MB-231 Cells

**FIGURE 4:**
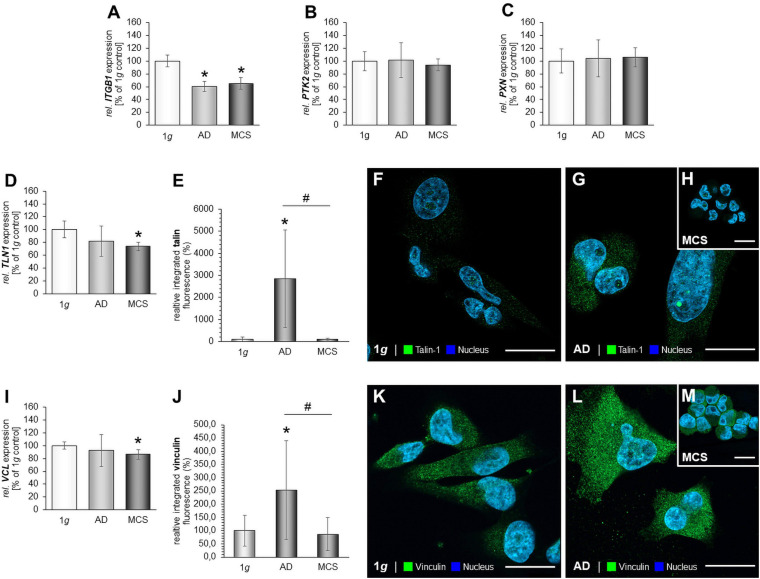
Focal adhesions in MDA-MB-231 cells. The gene expression of *ITGB1* **(A)**, *PTK2* mRNA expression **(B)**, *PXN* mRNA expression **(C)** of MDA-MB-231 cells exposed to the RPM. In addition, the gene expression of *TLN1* **(D)** and analysis of the fluorescence intensity of talin **(E)**, IFS of 1*g* control cells **(F)** and RPM-exposed MDA-MB-231 cells [AD cells: **(G)** and MCS: **(H)**] is given. Moreover, the *VCL* mRNA **(I)** and analysis of the fluorescence intensity of vinculin **(J)**, the IFS of vinculin are shown in 1*g* control cells **(K)** and RPM-exposed AD cells **(L)** and MCS **(M)**. *p < 0.05 1*g* vs. AD and/or MCS and #*p* < 0.05 AD vs. MCS.

We investigated integrin-β1 and measured a significantly down-regulated *ITGB1* gene expression in RPM-exposed AD and MCS samples (Figure 4A). The gene expression of focal adhesion kinase 1 (*FAK1*), also known as PTK2 protein tyrosine kinase 2 (*PTK2*), was not changed by RPM exposure in MDA-MB-231 cells (Figure 4B). In addition, the *PXN* mRNA was not altered in RPM-exposed MDA-MB-231 cells compared to 1*g* controls (Figure 4C).

Furthermore, the *TLN1* gene expression was not significantly altered in RPM-AD cells cultured under s-μ*g* conditions, but significantly reduced in MCS (Figure 4D). IFS revealed a cytoplasmic staining of talin-1. RPM-exposed AD cells exhibited an increase in talin-1 compared to 1*g* and MCS (Figures 4E–H). Furthermore, the MCS expressed talin-1 in an intensity comparable to 1*g* cells (Figure 4H).

In addition, the *VCL* gene expression was significantly down-regulated in MCS compared to 1*g* (Figure 4I). The IFS of vinculin showed a similar fluorescence intensity in the cytoplasm of the MDA-MB-231 MCS compared to 1*g*, but a significant increase in AD cells exposed to s-μ*g* (Figures 4J–M).

### The Impact of RPM-Exposure on Focal Adhesions in MCF-7 Cells

**FIGURE 5:**
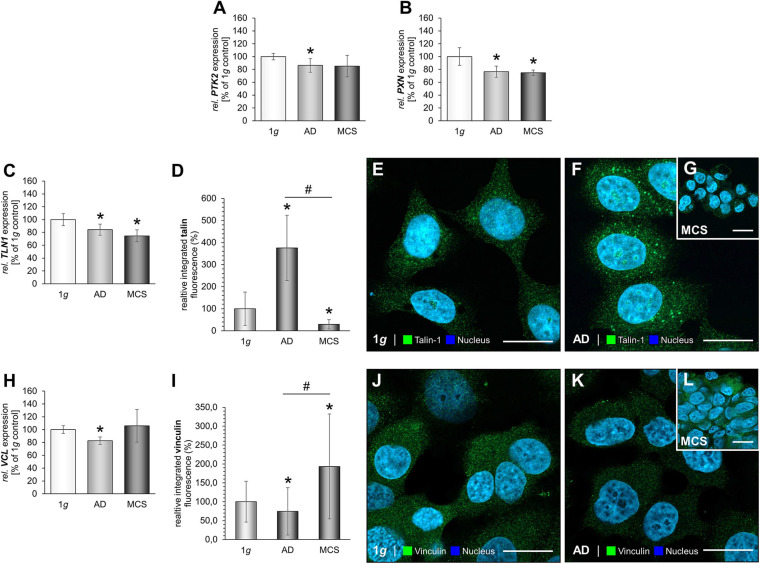
Focal adhesions in MCF-7 BCC. The *PTK2* mRNA expression **(A)**, *PXN* mRNA expression **(B)** of MCF-7 cells exposed to the RPM. In addition, the gene expression of *TLN1* **(C)** and analysis of the fluorescence intensity of talin **(D)**, IFS of 1*g* control cells **(E)** and RPM-exposed MCF-7 BCC [AD: **(F),** MCS: **(G)**] is given. *VCL* mRNA **(H)** and analysis of the fluorescence intensity of vinculin **(I)**, the IFS of vinculin are shown in 1*g* control cells **(J)** and RPM-exposed cells [AD: **(K),** MCS: **(L)**]. *p < 0.05 1*g* vs. AD and/or MCS and #*p* < 0.05 AD vs. MCS.

The next step was to examine focal adhesion molecules in MCF-7 BCC exposed to s-μ*g* conditions. The results are presented in Figure 5. *PTK2* mRNA expression was reduced in the AD group (Figure 5A). Furthermore, the gene expression of *PXN* was significantly down-regulated in both RPM groups (Figure 5B). Moreover, we focused on talin-1. The *TLN1* mRNA expression was significantly decreased in AD and MCS cells (Figure 5C). The IFS revealed a significant increase in the cytoplasmic fluorescence of talin in AD samples compared to MCS and 1*g* samples (Figures 5D–G). In MCS, the talin IF is significantly reduced compared with 1*g* (Figure 5G). Finally, the gene expression of *VCL* was not differentially displayed in MCS vs. 1*g*, but down-regulated in RPM-AD cells vs. 1*g* (Figure 5H). The IFS of vinculin is significantly reduced in AD cells compared to 1*g* cells, whereas the vinculin IF was elevated in MCS compared to 1*g* and AD (Figures 5I–L).

### The Impact of RPM-Exposure on E-Cadherin in MCF-7 Cells

**FIGURE 6:**
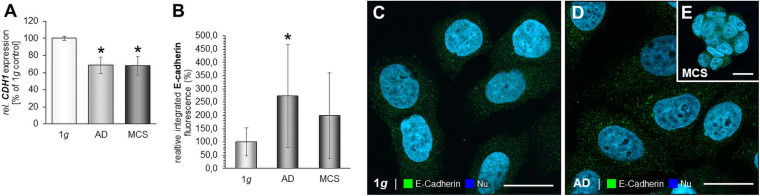
E-cadherin in microgravity-exposed MCF-7 BCC. **(A)** *CDH1* gene expression in MCF-7 BCC exposed to s-μ*g*. **(B)** Analysis of the fluorescence intensity of E-cadherin **(C)**, IFS of E-cadherin of 1*g* MCF-7 BCC RPM-exposed BCC [AD: **(D),** MCS: **(E)**]. *p < 0.05 1*g* vs. AD and/or MCS.

MCF-7 BCC exposed to the RPM showed a down-regulation of the *CDH1* gene in AD and MCS samples (Figure 6A). IFS revealed an elevated fluorescence intensity in AD cells compared to normal 1*g* control BCC (Figures 6B–E).

### Results of Gene/Protein Interactions (STRING Analysis)

**FIGURE 7:**
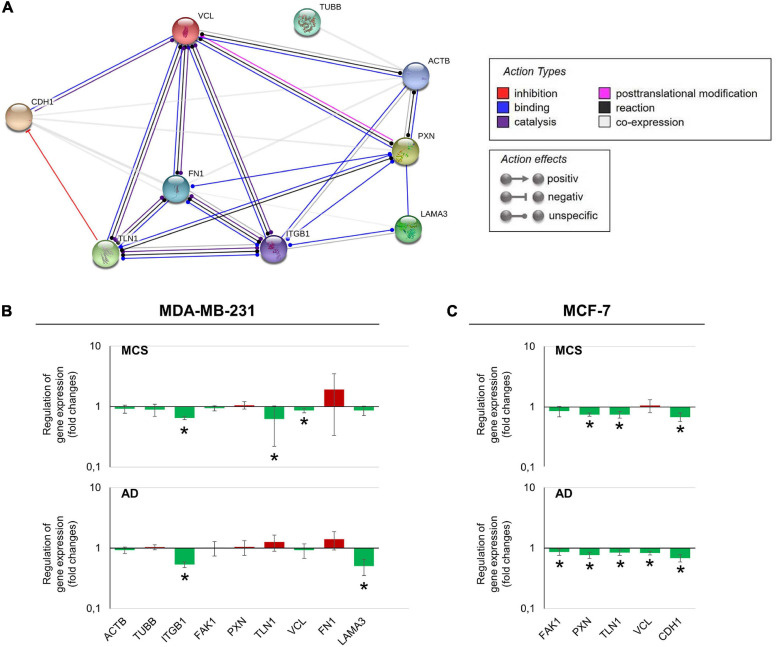
**(A)** Network of the functional interaction of genes and their products analyzed in this study. The analysis was performed by STRING (Search Tool for the Retrieval of Interacting Genes/Proteins, v11.0) provided by the STRING Consortium (available online: https://string-db.org/). The result is presented in the molecule action mode. Gene names are indicated. **(B)** Summary of the gene expression fold change measured by qPCR of 24 h RPM-exposed MDA-MB-231 samples. Results determined in AD and MCS in relation to 1*g* were given. The red color indicates up-regulated genes and the green color down-regulated genes. **(C)** Summary of the gene expression fold change measured by qPCR of 24-h RPM-exposed MCF-7 samples. The * indicates a significant change in fold change.

The various genes analyzed by qPCR were investigated with regard to their possible interactions and mutual expression dependence. A STRING/EMBL (European Molecular Biology Laboratory) analysis of these items represented in molecule action mode is shown in Figure 7A. It can be seen that the FA factors for which the expression pattern was analyzed regulate each other very strongly. The genes of interest (Figures 7B,C) were differentially regulated in s-μ*g* samples (AD and MCS). Figures 7B,C present a summary of the qPCR data, already described in Figures 2–6, and provide a comparable overview on the results. A closer look at the 24 h samples revealed that most genes involved in the focal adhesion molecule complex, especially the *CDH1* mRNA, were highly down-regulated in both AD and MCS groups of MCF-7 BCC. The cell adhesion molecule and extracellular matrix protein fibronectin was slightly elevated in AD and MCS. A down-regulation was observed for *LAMA3* in MDA-MB-231 cells.

### Vinculin/β-Catenin Signaling in MCF-7 Cells

**FIGURE 8:**
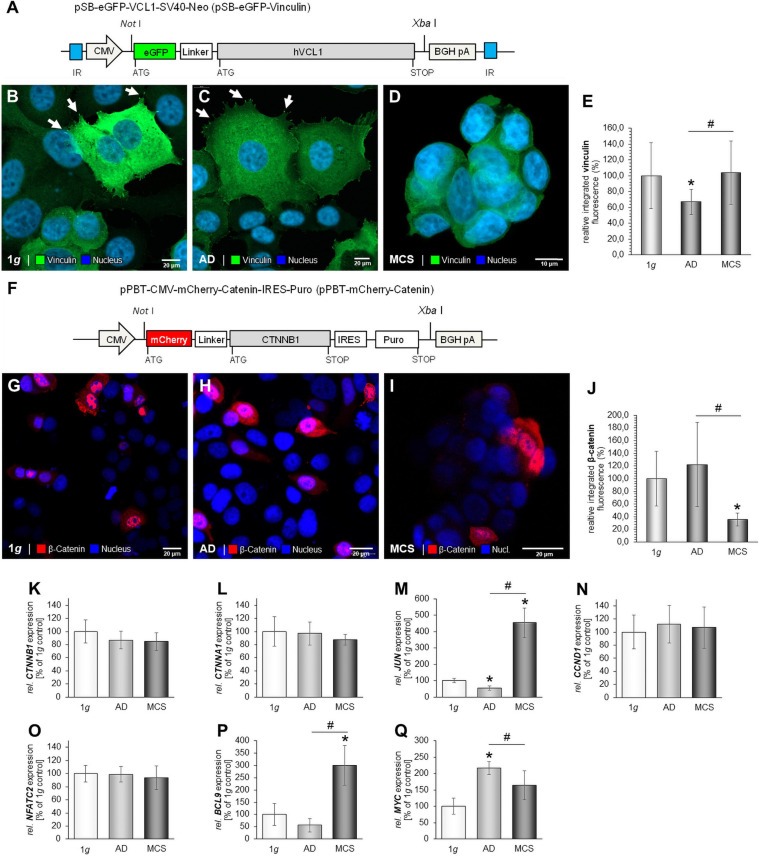
Stable expression of eGFP-vinculin in MCF-7 cells. Following co-transfection of pSB-eGFP-vinculin and pCMV-SB100X cells were cultured in medium containing G418 to allow growth of stably transfected cells only. **(A)** Graphic illustration of the pSB-eGFP-vinculin plasmid containing the coding sequence of the eGFP-vinculin fusion protein encoded from a CMV promoter. Restrictions used for cloning are indicated. BGH pA, bovine growth hormone polyadenylation site; CMV, cytomegalovirus promoter; IR, internal repeats. **(B)** Image of eGFP-vinculin in MCF-7 cells subjected to 1*g*. The white arrows show lamellipodia and vinculin focal adhesion dots. **(C)** Image of eGFP-vinculin in MCF-7 cells after RPM exposure. The white arrows indicate vinculin dots **(D)** Image of eGFP-vinculin in MCF-7 MCS following RPM-exposure. Scale bars 10 μm. Expression of mCherry-catenin in MCF-7 cells. MCF-7 cells were transfected with pPBT-mCherry-catenin and images were captured 48 h post transfection. **(E)** Analysis of the fluorescence intensity of eGFP-vinculin. **(F)** Graphic illustration of the pSB-eGFP-vinculin plasmid containing the coding sequence of the eGFP-vinculin fusion protein encoded from a CMV promoter. Restrictions used for cloning are indicated. BGH pA, bovine growth hormone polyadenylation site; CMV, cytomegalovirus promoter. **(G)** Image of MCF-7 cells expressing mCherry-catenin at 1*g*. **(H)** Image of RPM-exposed adherent MCF-7 cells expressing mCherry-catenin following incubation at μ*g*. **(I)** Image of MCF-7 MCS expressing mCherry-catenin following incubation at μ*g*. Scale bars 20 μm. **(J)** Analysis of the fluorescence intensity of mCherry-catenin. Gene expressions of *CTNNB1* **(K)**; *CTNNA1* **(L)**; JUN **(M)**; *CCND1* **(N)**; *NFATC2* **(O)**; *BCL9* mRNA **(P)** and *MYC* **(Q)**, MCF-7 cells exposed to the RPM. *p < 0.05 1*g* vs. AD and/or MCS and #*p* < 0.05 AD vs. MCS.

For stable expression and amenable live-cell visualization of vinculin in MCF-7 BCC, a Sleeping Beauty (SB) transposon-based vector containing a CNV-driven eGFP-hVCL1 cassette was constructed according to Nassef et al., 2019b; Figure 8A. This construct enables the synthesis of a fusion-protein containing the eGFP marker protein N-terminally tagged to vinculin. Following co-transfections with pSB-eGFP-vinculin and pCMV-SB100X, cells were incubated in medium containing G418 for selection of clones. Several colonies were obtained after inspection by fluorescence microscopy for validation of the expression level of eGFP-vinculin. Only few colonies were obtained when using an inactive SB transposase, showing that the eGFP-vinculin-positive clones results from SB-mediated insertion of the eGFP-hVCL1 cassette.

eGFP-vinculin is strongly expressed at 1*g* in transfected MCF-7 BCC. Stress fibers and lamellipodia are visible (Figure 8B, white arrows). A similar expression pattern was detected in RPM-exposed AD cells (Figure 8C). A prominent formation of stress fibers (see right side of the cells shown to the right in Figure 8C) is detectable. A uniform expression of eGFP-vinculin is seen in MCS (Figure 8D). Interestingly, the stress fibers had disappeared in BCC MCS (Figure 8D). Figure 8E gives the results of the analysis of the fluorescence intensity of eGFP-vinculin. The highest fluorescence intensity was measured in MCS and at 1*g*.

To visualize catenin in MCF7 BCC cells the cDNA fragment encoding human β-catenin N-terminally tagged with the mCherry marker protein were cloned into the pPBT-CMV-MCS-IRES-Puro plasmid (Figure 8F). Following transient transfection, the mCherry-catenin was expressed in the cytoplasm and the nucleus of MCF-7 cells grown under normal gravity conditions (Figure 8G). β-catenin is less located in the nuclei of MCS cells than of adherent cells after RPM exposure (Figures 8H,I). A non-uniform expression of mCherry-catenin is found in MCS (Figure 8I). The protein is accumulated at the outer cell membrane (Figure 8I). Figure 8J gives the results of the analysis of the fluorescence intensity of mCherry-catenin.

The *CTNNB1* mRNA was not significantly expressed in all three groups (Figure 8K). In addition, the gene expression of *CTNNA1* was not significantly altered (Figure 8L). The Wnt/β-catenin target gene *JUN* was down-regulated in AD samples and up-regulated in MCS (Figure 8M). The *CCND1* mRNA was not changed (Figure 8N). Furthermore, the *NFATC2* mRNA was not significantly altered after RPM exposure (Figure 8O).

The Wnt/β-catenin target genes *BCL9* and *MYC* were differentially expressed in RPM-exposed BCC (Figures 8P,Q). *BCL9* was significantly up-regulated in MCS compared to AD and 1*g* samples (Figure 8P). The *MYC* mRNA was significantly elevated in AD cells compared to 1*g* and MCS samples (Figure 8Q).

## Discussion

In this study, we focused on the changes induced in MDA-MB-231 and MCF-7 BCC when they were cultured under s-μ*g* conditions created by an RPM. The RPM, suggested also as ground-based facility by the European Space Agency, was chosen for our experiments because effects on various cells with respect to behavior and morphology seen in r-μ*g* are reproduced with good agreement (Wuest et al., 2015). Nevertheless, a final validation of our data is necessary in r-μ*g* to justify the term s-μ*g* in the context of our experimental approach. The MDA-MB-231 cell line is a good model for the basal-like or triple-negative form of BC (Chavez et al., 2010). This cell line was established from a pleural effusion of a patient with invasive ductal carcinoma and is E-cadherin-negative and expresses mutated p53. In contrast, the MCF-7 cell line is estrogen receptor (ER)-, progesterone receptor (PR)-, and E-cadherin-positive (Soule et al., 1973; Kopp et al., 2016), and it represents a model of a well-differentiated BC type.

The dynamic biological process of cell adhesion plays an important role in cell detachment and aggregation into 3D spheroids (Gumbiner, 1996). It has long been known that μ*g* influences cell-to-cell interactions and alters the cytoskeleton, integrins, and ECM components of mammalian cells (Buravkova et al., 2005; Aleshcheva et al., 2016; Hader et al., 2017).

Various researchers have demonstrated that the adherence behavior of human cells changes when they are exposed to μ*g* conditions (Dittrich et al., 2018; Zhao et al., 2018; Deng et al., 2019; Nassef et al., 2019a; Romswinkel et al., 2019). In particular, cell adhesion changes are involved in the scaffold-free formation of MCS (Riwaldt et al., 2015a; Svejgaard et al., 2015; Dittrich et al., 2018; Buken et al., 2019). The two cell adhesion molecules ICAM-1 and VCAM-1 are significantly altered by μ*g* in different mammalian cells (Paulsen et al., 2015; Riwaldt et al., 2015b; Tauber et al., 2017; Buken et al., 2019). Their specific involvement in 3D growth was suggested for thyroid cancer cells after the CellBox-1 space mission (Riwaldt et al., 2015a).

### Changes in the Cytoskeleton and Extracellular Matrix

It is well known that μ*g* induces a disorganization in the network of microfilaments, intermediate filaments, and microtubules in various cell types (Vorselen et al., 2014). Several studies have revealed that the formation of the cytoskeleton is highly sensitive to alterations in gravity and shown that altered gravity conditions have an enormous impact on the cytoskeleton and the differential expression of genes related to it (Vassy et al., 2001; Uva et al., 2002; Ulbrich et al., 2011; Aleshcheva et al., 2013, 2015; Corydon et al., 2016a).

In this study, we observed that the gene expression of *ACTB* was not differentially altered in MDA-MB-231 cells after 24 h of RPM exposure. This is in accordance with earlier results obtained from MCF-7 BCC cultured for 24 h on the RPM (Kopp et al., 2016). In parallel, the *TUBB* gene expression was not altered in adherently growing RPM-exposed MDA-MB-231 cells compared with 1*g* samples. *TUBB* remained unchanged in MCS samples. This finding is different to the *TUBB* gene expression of MCF-7 BCC, FTC-133 thyroid cancer cells, and normal thyroid cells exposed to s-μ*g* (Kopp et al., 2015, 2016), where a downregulation of the *TUBB* gene was observed in MCS of these cell types. This difference may be explained by the low differentiation of the MDA-MB-231 BC type.

In addition, we studied the expression of fibronectin. In general, we found a slight but insignificant trend for an increase in *FN1* at the mRNA level when MDA-MB-231 cells were exposed to the RPM. Interestingly, the IIF staining revealed an increase of fibronectin protein in AD and MCS cells. In general protein and mRNA abundance, although derived from the same tissue or cell type, is usually in a non-linear relationship as multiple processes beyond transcript concentration contribute to protein abundance. Protein abundance modulation through the binding to regulatory elements (e.g., micro-RNAs), protein’s half-life, autophagy, protein synthesis delay by physical distance, availability of involved actors and feedback regulatory mechanisms usually leading to a reduced correlation between mRNA and protein levels.

This protein data fits with the results of an earlier study on human fetal osteoblasts exposed to the RPM, where the cells showed elevated *FN1* gene expression and fibronectin protein content after a seven-day period of RPM exposure (Mann et al., 2019). In addition, the *FN1* gene expression was not changed in MCF-7 spheroids after 24 h of RPM-exposure (Kopp et al., 2016).

*LAMA3* mRNA was reduced in AD samples, but not differentially expressed in MDA-MB-231 MCS samples compared with 1*g* controls. This is in contrast to the data obtained from MCF-7 cells, where *LAMA3* was significantly up-regulated in MCS (Kopp et al., 2016). This finding may be due to the formation of gland-like MCS when MCF-7 cells were exposed to the RPM for 5 days (Kopp et al., 2016). Laminin is a component of the basal lamina of a cell and influences the biological processes differentiation, migration and adhesion. This might explain the high amount of *LAMA3* mRNA in MCS, where a high grade of differentiation and changes in the extracellular matrix components occur to form the 3D glandular structures.

### The Focal Adhesions of BCC Grown in Simulated Microgravity

Furthermore, we focused on the FAs of MDA-MB-231 and MCF-7 BCC exposed to the RPM. FAs are subcellular structures mediating cellular signaling processes as a reaction to ECM adhesion and are known as mechanical linkages to the ECM. FAs are key players in gravisensing and the detachment of cells and following aggregation into 3D assemblies. This aggregation occurs through changes in the mechanical loading conditions in an s-μ*g* environment, among other pathways (Geiger et al., 2009; Romswinkel et al., 2019).

Vinculin has an impact on cell–matrix adhesion and intercellular junctions and plays an important role in mechanotransduction with integrins at FA sites (Spanjaard and de Rooij, 2013). Vinculin interacts with talin, integrins, and actin, and thus influences cellular migration and focal adhesion. Vinculin is a mediator of cellular and extracellular signals. The gene expression of *VCL* was reduced in the MCS of MDA-MB-231 cells compared to 1*g* (Figure 4I). IFS of vinculin revealed an increase of the fluorescence intensity in AD samples and no change in MDA-MB-231 MCS.

The other important FA component investigated in this study is the cytosolic and mechanosensitive protein talin. Its major task is to link integrins directly and indirectly *via* vinculin to the cytoskeleton. Talin-1 is involved in cell adhesion, progression, extravasation, and *trans-*endothelial migration in cancer. Integrins bind to talin, and then talin connects to vinculin to influence the process of cell adhesion. Finally, integrin receptors promote the attachment of adherent growing cells to the ECM. In this context, talin acts as mechanotransductor. In triple-negative MDA-MB-231 BCC, the *TLN1* gene expression in AD was not significantly changed, but reduced in MCS. The IFS of talin-1 revealed an increase in talin-1 protein in AD samples compared to 1*g* and MCS in TNBC (Figure 4E). In MCF-7 cells, the *TLN1* gene expression was significantly reduced in AD and MCS cells compared to 1*g* (Figure 5C). As talin carries the mechanical force in ECM and cell adhesion, the reduced expression of talin would indicate less cell adhesion and cell detachment in the s-μ*g* environment.

The cell surface receptor and cell adhesion molecule integrin-β1 is encoded by the *ITGB1* gene. We found a down-regulation of *ITGB1* in MDA-MB-231 cells exposed to s-μ*g* (Figure 4A). Integrin-β1 links the actin cytoskeleton with the ECM, and, thus, signaling transduction is possible between the ECM and the cytoplasm. In addition, integrin-β1 modulates gene expression within the cell and is a key player in mechanotransduction (Katsumi et al., 2004; Legate et al., 2009). Integrin-β1, talin, vinculin, fibronectin, and laminin play key roles in focal adhesion processes and can give hint that focal adhesion is less in MCS. These findings are in agreement with the results of a recently published paper (Monti et al., 2021). The adhesion process is very important for the survival strategy of cancer cells to antagonize cell death when the cells are exposed to the RPM. The cytoskeleton, integrins and focal adhesion factors are involved in anti-apoptotic strategies, depending on the cell type (Monti et al., 2021). No signs of apoptosis were found in MCF-7 cells after a 24-h RPM-exposure, which was paralleled by a reduction of vinculin and integrin-β1 in MCS (Monti et al., 2021).

Furthermore, the focal adhesion kinase [FAK/protein tyrosine kinase 2 (PTK2)] is involved in mechanosensing and transduction, proliferation, and migration; it links the ECM and the cytoskeleton (McLean et al., 2005). *FAK1/PTK2* gene expression was not changed in all MDA-MB-231 groups (Figure 4B). The protein paxillin is encoded by the *PXN* gene. Paxillin is expressed at FA and its function is to adhere human cells to the ECM. Other FA proteins can bind to paxillin, like FAK1 or vinculin. In MCF-7 cells, the *PXN* mRNA in AD and MCS and the corresponding proteins were reduced as compared to 1*g* (Figure 5B). In MDA-MB-231 cells, *PXN* gene expression was unchanged (Figure 4C). These findings fit to the results obtained for integrin-β1, which were similarly regulated. This underlines their close interaction when BCCs were exposed to s-μ*g*, leading to cell detachment and the formation of 3D spheroids, where the expression level of FA components can regulate migration and 3D aggregation.

### Interaction Network of Selected Genes Evaluated by STRING Analysis

The STRING analysis revealed several interactions between the selected factors. *FN1, VCL, TLN1*, and *ITGB1* are indicated as dominant target genes, as many arrows point to their icons (Figure 7A). Fibronectin was found to be the central target gene. There exist interactions between vinculin and fibronectin. The *FN1* gene expression was unaltered in MDA-MB-231 cells (Figure 3A). The *FN1* gene expression was also not changed in MCF-7 BCC as published earlier (Kopp et al., 2016). The *VCL* mRNA was down-regulated in RPM-exposed MDA-MB-231 MCS cells (Figure 4I). In MCF-7 cells, the *VCL* mRNA was significantly reduced in AD cells. Differences between BC cell types may be due to the different characteristic features of the two breast BC types.

Fibronectin is elevated in podocytes by mechanical stress (Kliewe et al., 2019). Fibronectin KO podocytes showed significantly down-regulated FA molecules (talin, vinculin, and paxillin) as well as a reduction in cell spreading, indicating an important role of fibronectin in adhesion (Kliewe et al., 2019). Fibronectin plays a key role in the adaptation of podocytes to mechanical stress. This finding supports the hypothesis that the interaction of FN1 with vinculin is an adaptive mechanism to protect μ*g*-exposed BCC from mechanical stress. FN1 also interacts with talin and paxillin. Talin links intracellular networks with the ECM *via* its connection with the actin cytoskeleton and membrane integrins (Haining et al., 2016). Talin exposes new recognition sites when undergoing force-induced mechanical unfolding, and it can bind to and recruit cytoskeletal proteins that are involved in mechanotransduction and MCS formation (Haining et al., 2016). Moreover, paxillin and fibronectin are involved in embryonic developmental events, possibly due to paxillin-mediated modulation of fibronectin-regulated focal adhesion dynamics and organization of the membrane cytoskeletal structures. Thus, paxillin regulates cell migration and spreading of BCC (Hagel et al., 2002). In addition, VCL interacts with CDH1. The cell adhesion glycoprotein E-cadherin (CDH1) is commonly inactivated and reduced in progressive breast tumors. In MCS of MCF-7 cells, it was down-regulated after 14 days of RPM exposure (Sahana et al., 2018). Vinculin is involved in the establishment or regulation of the cadherin-based cell adhesion complex. This process is mediated by a direct interaction with β-catenin (Hazan et al., 1997). The loss of cell–cell adhesion is involved in cancer invasion and metastasis. It has been shown that vinculin impacts metastasis and prognosis in several tumors (Li et al., 2014; Zhang et al., 2019).

As demonstrated in Figure 4I, the *VCL* gene expression levels were reduced in MCS of MDA-MB-231. In addition, *VCL* mRNA was significantly down-regulated in MCF-7 AD cells. These findings were also confirmed in highly metastatic CRC cell lines and metastatic tissues (Li et al., 2014).

The *ITGB1* gene expression was significantly reduced in MCS of MCF-7 cells (Kopp et al., 2016). This was also measured in MDA-MB-231 cells, where the *ITGB1* mRNA was clearly down-regulated in AD and MCS cells (Figure 4A). Integrin-β1 is a membrane protein and is linked to the ECM with the cytoskeleton; it is also capable of transmitting signals (Yeh et al., 2012).

The STRING analysis revealed an interaction between fibronectin and integrin-β1, both playing a central role in 3D formation (Buken et al., 2019). When integrin-β1 is activated and forms a heterodimer with an appropriate integrin-α, integrin-β1 can bind to fibronectin, laminin, and other ECM components *via* the extracellular domain (Lessan et al., 1999). In addition, integrin-β1 can be activated by binding talin and kindlin *via* its cytoplasmic domain (Meves et al., 2013). This binding signal will be transferred to the cytoskeleton and the FA complex *via* vinculin and actin (Gingras et al., 2010).

### Vinculin and β-Catenin Signaling in RPM-Exposed MCF-7 Cells

It is known that vinculin regulates the cell surface E-cadherin expression by regulating β-catenin (Peng et al., 2010). In addition, the loss of vinculin and membrane-bound β-catenin promotes metastasis in colorectal cancer (Li et al., 2014) and may also play a role in progression of other cancer types. The loss of cell adhesion is important for spheroid formation and also for metastasis. Vinculin is a key adhesion-related protein and is involved in metastasis in various tumors (Li et al., 2014).

As shown in Figure 6, RPM-exposure of MCF-7 cells resulted in a down-regulation of *CDH1*. Therefore, we focused in detail on vinculin and β-catenin signaling in MCF-7 cells. Unfortunately, MDA-MB-231 cells do not express E-cadherin (Eslami Amirabadi et al., 2019), and, therefore, we used the MCF-7 cell line for this study.

Live cell imaging of vinculin/β-catenin transfected MCF-7 cells exposed to the RPM revealed that vinculin was distributed throughout the cytoplasm. The protein was detectable at adhesive membrane areas. It is known to mediate the interactions of integrins and the actin cytoskeleton (Bays and DeMali, 2017). Vinculin regulates the adhesion process by interacting with actin. The connection of vinculin to F-actin is important in regard to its role in cell–matrix adhesion. Changes in the vinculin–F-actin interaction can impact morphology, stiffness, adhesion and migration of the cells (Ezzell et al., 1997).

β-catenin (mCherry) was located in the nucleus and cytoplasm in all adherent cells (Figure 8; 1*g*, RPM-AD). In MCS, β-catenin was detectable in the membranes. β-catenin is a known cadherin-associated protein and an important cell adhesion regulator. Furthermore, β-catenin is a transcriptional co-activator in the nucleus and involved the canonical Wnt signal transduction pathways. Therefore, we have focused on Wnt/β-catenin signaling and on the gene expression of several target genes such as *BCL9, MYC, JUN* and *CCND1* (cyclin D1). Both, *CTNNA1* and *CTNNB1*, mRNAs were not significantly altered by gravitational unloading using the RPM. A similar result for the *CTNNB1* gene expression was obtained when low-differentiated follicular thyroid cancer cells (FTC-133 cell line) were exposed for 4 h or 3 days to the RPM (Melnik et al., 2020). Additional studies using cells providing stable expression of β-catenin may further support the results obtained in transiently transfected MCF-7 cells.

It was reported that mechanical unloading of wildtype mice caused a decrease in Wnt/β-catenin signaling activity accompanied by upregulation of Sost, and it is involved in osteoporosis (Lin et al., 2009). Furthermore, the β-catenin location is sensitive to s-μ*g* in osteoblasts (Yin et al., 2018). The nuclear factor of activated T cells (NFAT) is involved in proliferation, angiogenesis, migration in various cancer types (Werneck et al., 2011) and is associated with epithelial–mesenchymal transition (EMT) in breast cancer (Quang et al., 2015). Therefore, we studied the expression of *NFATC2* in our experimental setting. We measured that both *NFATC2* and *CCND1* (cyclin D) were not significantly changed after RPM exposure in MCF-7 cells. An earlier study investigated endothelial cells (EA.hy926 cell line) during a parabolic flight mission (Wehland et al., 2013). The gene expression of *CCND1* was reduced when EA.hy926 cells were studied under vibration (Wehland et al., 2013). No change in the gene expression was measurable when the cells were exposed to hypergravity conditions (Wehland et al., 2013). After one parabola (P) and 31P (real microgravity), the *CCND1* gene was up-regulated (Wehland et al., 2013).

Interestingly, we measured a significant increase in the *BCL9* (B-cell lymphoma 9) and *JUN/c-JUN* (Jun proto-oncogene, AP-1 transcription factor subunit) gene expression of the MCS compared to the 1*g* and the RPM-AD group. The oncogene *BCL9* functions as a transcriptional co-activator of the Wnt/β-catenin signaling pathway in cancer. *BCL9* plays a key role in tumor progression and remodeling of the tumor microenvironment (Jiang et al., 2020). Its upregulation in RPM-exposed samples hints to its involvement in spheroid formation. Little is known about *JUN* in microgravity-exposed cells. A recent study (Shi et al., 2021) investigated the effects of μ*g* on macrophage differentiation from hematopoietic progenitor cells (HPCs) and demonstrated that a 12-day RCCS exposure of these cells reduced the amount of p-JUN in the RCCS-exposed group.

Furthermore, we investigated the *MYC* (c-myc) gene expression. *MYC* was significantly elevated in RPM-AD cells as compared to the 1*g* and MCS group. This is an interesting finding which was also observed *in vivo* in mice exposed to a 30-day spaceflight (Radugina et al., 2018). Radugina et al. demonstrated the nuclear immunolocalization of c-Jun and c-Myc proteins, indicating their sensitivity to μ*g*. Future research is necessary to gain more information about the impact of μ*g* on cancer growth and the involvement of the Wnt/β-catenin signaling pathway.

In summary, a 24 h RPM exposure of triple-negative MDA-MB-231 and MCF-7 BCC induced changes in growth, the cytoskeleton, the extracellular matrix, and focal adhesions. As demonstrated in Figure 1, multicellular tumor spheroids of both cell lines showed a fast migration behavior and clear invasion potential when co-cultured with EA.hy926 endothelial cells. After a 24 h RPM-cultivation, multiple round compact MCS and in parallel adherent cells were visible in the cell culture supernatant of both cell types. The 3D aggregates will be used in future studies to examine the effects of anticancer drugs. MCS closely mimic the form and micromilieu of a metastasis. In this study, MDA-MB-231 BC MCS cells grown under conditions of s-μ*g* exhibited a differential regulation of the expression of ECM, and FA genes, such as *ITGB1, VCL* and *TLN1*. RPM exposure of MCF-7 BC MCS cells resulted in significant changes in the gene expression of *PXN, TLN1*, and *CDH1*, and the WNT-β-catenin target genes *BCL9, JUN* and *MYC*. The focal adhesion complex as well as cell adhesion molecules and the cytoskeleton are important gravisensors in μ*g*.

Taken together, the overall gene expression pattern is only slightly modified by RPM-exposure as previously evidenced (Po et al., 2019). Facing an essential maintenance of an overall coherence in gene expression pattern, the only few genes that are modified by microgravity are those involved in growth, the cytoskeleton and mechanotransduction.

In conclusion, BCC (MCF-7 and MDA-MB-231 cells) were exposed to s-μ*g* conditions generated by an RPM. During cell culture on the RPM, the cells transformed from a 2D monolayer into tissue specific multicellular *in vitro* 3D spheroids. The RPM-exposed cells revealed alterations in cytoskeletal proteins, as well as changes in ECM components and FA factors. A 24-h RPM-exposure induced a significant down-regulation of *ITGB* and *LAMA3* mRNAs in adherent cells and in MCS of the MDA-MB-231 cell line. Moreover, the *VCL* mRNA was significantly reduced in MDA-MB-231 MCS cells. In contrast, the gene expression of *FAK1, PXN, TLN1, VCL*, and *CDH1* was significantly down-regulated in adherent MCF-7 cells cultured for 24 h on the RPM. In addition, *PXN, TLN1* and *CDH1* were down-regulated in MCS, whereas *VCL* and *LAMA3* mRNAs were not changed in MCF-7 cells.

Overall, this study provides novel knowledge about the complexity of adherence, migration and invasion behavior of human BCC spheroids engineered with s-μ*g* technology. Further investigations targeting FA proteins and genes in BCC cultured on an RPM will hopefully, indicate novel targets that are interesting future candidates for cancer therapy.

## Data Availability Statement

The datasets analyzed for this study can be provided by contacting the corresponding author.

## Author Contributions

DG and TC: conceptualization. SKo and JS: methodology. SKo: software. DG, TC, MW, SKo, and MK: validation. DM, SKa, SKo, JS, and MK: formal analysis. JS: investigation. BR, MI, and DG: resources. DG, JS, and TC: writing—original draft preparation. DG, TC, MK, and MW: writing—review and editing. MK and SKa: visualization. DG: supervision and project administration. DG and MI: funding acquisition. All authors have read and agreed to the published version of the manuscript.

## Conflict of Interest

The authors declare that the research was conducted in the absence of any commercial or financial relationships that could be construed as a potential conflict of interest. The reviewer RH declared a past co-authorship with one of the authors, DG.

## Footnotes

1 https://fiji.sc/
